# Dietary Antioxidants in Coffee Leaves: Impact of Botanical Origin and Maturity on Chlorogenic Acids and Xanthones

**DOI:** 10.3390/antiox9010006

**Published:** 2019-12-20

**Authors:** Ângelo Monteiro, Silvia Colomban, Helena G. Azinheira, Leonor Guerra-Guimarães, Maria Do Céu Silva, Luciano Navarini, Marina Resmini

**Affiliations:** 1 Department of Chemistry, Queen Mary University of London, Mile End Road, London E1 4NS, UK; 2 Illycaffè S.p.A., via Flavia 143, 34100 Trieste, Italy; silvia.colomban@illy.com (S.C.); luciano.navarini@illy.com (L.N.); 3 Centro de Investigação das Ferrugens do Cafeeiro, Instituto Superior de Agronomia, Universidade de Lisboa, 2784-505 Oeiras, Portugal; hgazinheira@isa.ulisboa.pt (H.G.A.); leonorguimaraes@edu.ulisboa.pt (L.G.-G.); mariaceusilva@isa.ulisboa.pt (M.D.C.S.); 4 Linking Landscape, Environment, Agricultural and Food, Instituto Superior de Agronomia, Universidade de Lisboa, 1349-017 Lisboa, Portugal

**Keywords:** antioxidants, alkaloids, chlorogenic acids, coffee leaves, leaf development stages, mangiferin, xanthones

## Abstract

Natural polyphenols are important dietary antioxidants that significantly benefit human health. Coffee and tea have been shown to largely contribute to the dietary intake of these antioxidants in several populations. More recently, the use of coffee leaves to produce tea has become a potential commercial target, therefore prompting studies on the quantification of polyphenols in coffee leaves. In this study a variety of coffee leaf species, at different development stages, were analyzed using ultra-high pressure liquid chromatography. The results demonstrate that both the botanical origin of the samples and their maturity influence significantly the concentration of the antioxidants; for total chlorogenic acids a two-fold difference was found between different species and up to a three-fold variation was observed between young and mature leaves. Furthermore, the range of concentrations of chlorogenic acids in young leaves (35.7–80.8 mg/g of dry matter) were found to be comparable to the one reported for green coffee beans. The results provide important data from which potential new commercial products can be developed.

## 1. Introduction

Natural polyphenols, one of the most important groups of dietary antioxidants, include a wide variety of chemical compounds, such as flavones, flavonols, isoflavones, and phenolic acids, including chlorogenic acids (CGAs) [1]. All these secondary metabolites are widely found in fruits, vegetables, grains, and cocoa, as well as coffee, tea, and wine [2]. These phytochemicals are of great interest to nutritionists, food scientists and consumers due to their demonstrated impact on human health, especially in the prevention of degenerative and cardiovascular diseases, and also metabolic disorders [3]. Coffee and tea, in particular, have been found to play a key role in the polyphenol dietary intake of consumers, with the consumption of these drinks responsible in some cases for >50% of polyphenols intake [4,5,6].

Coffee, with a global production of 158.6 million 60-kg bags in 2017 and an export value of 32.7 billion dollars [7], is the second most popular beverage worldwide after water. The chemical composition of the beans of the two most commercially exploited coffee species, *Coffea arabica* Linn. and *Coffea canephora* Pierre ex A. Froehner, has been extensively studied because of their relevance to cup quality, product traceability, authenticity, and safety. Significant research was carried out to understand the chemistry of green coffee beans with the aim of improving quality [8]. Coffee beans are not the only source of antioxidants in the coffee plant.

Coffee leaves have been commonly used to make infusions in many coffee producing countries such as Ethiopia, South Sudan, and Indonesia. The beverage is believed to help on the treatment of several disorders, for instance anemia, intestinal pain, and fever [9]. In 2013, Wize Monkey, a Canadian company, started selling coffee leaf tea all over the world, with great success, confirming the growing interest in leaves as a commodity. For this reason, the study of the chemical composition of coffee leaves, from which tea is made, is now a priority. A number of studies have been done to identify bioactive compounds present in coffee leaves [10,11,12,13]. The results identified three main classes of bioactive compounds, namely alkaloids, chlorogenic acids and more recently xanthones [13]. However, with one exception [11] there is a significant lack of data in literature regarding the effect of aging and interspecific variation on the antioxidants’ concentration.

Chlorogenic acids (CGAs) are the main class of polyphenols in coffee beans, where they can be found in large quantities [14]. Metabolic fate and bioavailability of these compounds have been recently investigated, and were found to be absorbed and excreted to a much greater extent than many other dietary flavonoids and phenolic compounds [15]. The high accumulation of CGAs in the coffee leaves of different species has been previously reported, in which 5-caffeoylquinic acid (5-CQA) and 3,5-dicaffeoylquinic acid (3,5-DiCQA) were found to be the most abundant chlorogenic acids [11,13,16,17]. 

Xanthones, which are famously known by mangostin and mangiferin, possess valuable biological activities with a great potential for pharmacological applications [18]. In particular, mangiferin can be mainly found in different tissues of *Mangifera indica* (mango tree) and *Cyclopia genistoides* (honeybush), which are widely used in traditional medicine [19]. Coffee leaves have been found to contain an amount of mangiferin comparable to *M. indica* leaves [20]. Recently, the accumulation of mangiferin has been demonstrated in the leaves of several coffee species, suggesting that this maybe an excellent natural source of this xanthone [11,12].

The third class of bioactive compounds present in coffee leaves is alkaloids, particularly xanthines and trigonelline, whose biological properties have been studied [21,22]. Caffeine, the most widely consumed psychostimulant worldwide, exerts a wide range of effects on the human body and for this reason metabolism and pharmacokinetics have been extensively studied in the past and revised recently [21]. Caffeine and its analogues may also contribute to the overall antioxidant properties of coffee [23,24]. The presence of caffeine in coffee leaves makes the coffee leaf tea an alternative to the traditional tea beverage, in terms of central nervous system stimulation [10], in addition to other bioactive flavonoids including rutin, quercetin and kaempferol glycosides which have been recently reported [13].

The potential health benefits of coffee-leaf tea as beverage [25,26] is of great interest to the coffee community, as a mean of adding value to waste biomass and also to coffee species with very low beans yield and, such as *Coffea racemosa* Lour. and *Coffea eugenioides* S. Moore. This work focused on the profiling and comparison of several coffee leaf species at different development stages, with the quantification of the major bioactive compounds, i.e., alkaloids, chlorogenic acids and xanthones, done by ultra-high-performance liquid chromatography (UHPLC). The results are expected to provide a phytochemical profile that will aid further applications of this source of active compounds in the food industry.

## 2. Materials and Methods 

### 2.1. Chemicals

For quantitative purposes primary reference standards were used where possible: 3-caffeoylquinic acid (3CQA), 4-caffeoylquinic acid (4CQA), 5-caffeoylquinic acid (5CQA), 3,4-dicaffeoylquinic acid (3,4-diCQA), 3,5-dicaffeoylquinic acid (3,5-diCQA), and 4,5-dicaffeoylquinic acid (4,5-diCQA) were obtained from PhytoLab GmbH & Co. KG (Vestenbergsgreuth, Germany); caffeine (Ph. Eur. Grade), theobromine, mangiferin trigonelline hydrochloride (analytical grade) methanol and ethanol (HPLC grade) from Sigma-Aldrich Chemie GmbH (Steinheim, Germany). Qualitative identification was performed using synthetized standards of FQAs and pCoQAs, not commercially-available. Sodium metabisulfite was obtained from VWR International (Fountenay-sous-Boiscedex, France). All solutions were made with milliQwater system (Millipore, Molsheim, France) and methanol (7:3 *v:v*).

### 2.2. Taxon Sampling and Plant Material

The species, accession and origin of the coffee plants used are indicated in Table S1. Leaves were collected from plants of *C. arabica* cv. Bourbon maintained in greenhouse conditions (private collection, Illycaffè, Trieste, Italy) during the month of July 2017. A selection was made between young leaves (fully expanded leaves of the terminal node) and mature leaves (fully expanded leaves of 2nd pair from branch apex), medium sizes are reported in Table S1. *C. canephora*, *C. eugenioides* and *C. racemosa* plants were maintained at Centro de Investigação das Ferrugens do Cafeeiro (CIFC) in greenhouse conditions.

### 2.3. Preparation of Plant Material

Fully expanded leaves of the terminal node (young) and from the second pair (mature) were collected, measured and frozen in liquid nitrogen before being freeze-dried (Ilshin, Lab Co. Ltd., Dongducheon, South Korea) to a constant weight. Dried samples were ground by blending to a fine homogenous powder in a Reutsch MM400 Mixer Mill prior to extraction.

### 2.4. Coffee Leaf Extraction

Extraction of phytochemicals was carried out as described: 200 mg of plant material (lyophilized leaves ground with a mixer mill) was sonicated for 30 min. at room temperature (35 kHz, Bandelin sonorex, Berlin, Germany) in 4 mL of EtOH/H_2_O (50:50, *v/v*), containing sodium metabisulfite (1 g/L). After centrifugation (5 min, 8602× *g* RCF at 10 °C, Allegra 64R Centrifuge, Beckman Coulter, Indianapolis, IN, USA) the ethanolic extract was collected and filtered (Phenex, NY 0.20 μm porosity, Phenomenex, Torrance, CA, USA) and kept at 4 °C before analysis. Each extraction was performed at least in duplicate. Each sample was characterized by its mean content of alkaloids, chlorogenic acids and xanthones, expressed as mg/g of leaves (dry weight).

### 2.5. Validation of the Ultra-High Performance Liquid Chromatography-DAD method

The evaluation of the limit of detection (LOD) and limit of quantitation (LOQ) for the individual compounds were calculated based on a signal-to-noise (S/N) ratio >3 (LOD) and S/N >10 (LOQ), using the following formulas:

LOD = 3 × c / (S/N)(1)

LOQ = 10 × c / (S/N)(2)

where c = concentration.

The repeatability of the peak areas was check at all the concentrations for each compound by calculating the relative standard deviation (RSD) and percent bias value for 5 replicates over the course of 3 days as follows: RSD (%) = (standard deviation/mean) × 100 and Bias (%) = [(calculated concentration − theoretical concentration)/theoretical concentration] × 100.

### 2.6. Description of Ultra-High Performance Liquid Chromatography-DAD

Quantitative analyses were carried out using a 1290 UHPLC system (Agilent Technologies, Santa Clara, CA, USA) equipped with a degasser, quaternary pump, column thermostat and diode array detector (DAD) operating at 254, 258, 273, 305, and 324 nm. A Kinetex^®^ 2.6 μm XB-C18 100 Å, LC column 30 × 2.1 mm (Phenomenex, Torrance, CA, USA) was used at room temperature. The elution system (0.4 mL min^−1^) involved two filtered solvents, namely acetonitrile (solvent A) and 0.1% formic acid in water (solvent B) with the following gradient: 0 min, 99% solvent B; 0–6 min, 90%; 6–10 min, 60%; 10–11 min, 99%; isocratic, 99% until 14 min. The injection volume was 2 µL. The identification of alkaloids, chlorogenic acids and xanthones were achieved by comparison of specific retention times of standard solutions; quantitative determination was performed using calibration curves of standards. Standards stock solutions were prepared in MeOH:H_2_O (3:7) over the concentration range 1 and 450 mg/L. Different diluted solutions were prepared from stock solutions with water. Stock and diluted solutions were kept refrigerated at 4 °C before use.

### 2.7. Description of Ultra-High Performance Liquid Chromatography Electrospray Ionization Mass Spectrometry

UHPLC-ESI-MS analysis was conducted on an Agilent 1290 HPLC (Santa Clara, CA, USA), coupled to a Sciex Triple Quad 4500 (Farmingham, MA, USA). Chromatographic separation of ethanol extracts was conducted using a column of the same type, and dimensions as for analytical UHPLC, with identical gradient elution. MS Electrospray Ionisation Source was operating in negative mode, acquiring in Multiple Reaction Monitoring (MRM). Operating conditions were optimized using a 5-CQA standard solutions. The source temperature was 350 °C. Specific compounds transitions were confirmed based on literature data and comparison with reference standard solutions. Taking into consideration that, according to the literature [27] *cis* isomers show the same fragmentation pattern of the corresponding trans isomers, the presence of possible *cis* isomers was confirmed by comparison of specific fragmentation of the UV treated solutions.

### 2.8. Statistical Analysis

Data were analyzed by one-way analysis of variance (ANOVA) using XLStat 2018 (Addinsoft, Bordeaux, France). Significant differences among samples were compared using Tukey’s tests. Data were expressed as means ± SD and *p* < 0.05 represents statistically significant difference.

## 3. Results and Discussion

### 3.1. Validation of the UHPLC-DAD Method

In this work, ultra-high pressure liquid chromatography (UHPLC) was preferred over high pressure liquid chromatography (HPLC) as the analytical tool, because it is more economical and offers higher sensitivity. UHPLC uses shorter and narrower columns filled with small-sized particles (<3 µm) in combination with very high pressure, resulting in lower volumes of solvents used, shorter analysis time, increased sensitivity, and excellent resolution [28]. The use of the diode array detector (DAD) coupled to the UHPLC ensures the clear identification of each component. HPLC coupled with a DAD is the most reported method for the quantification of the main secondary metabolites present in coffee leaves [11,12,13], in which each run averaging over 25 min. The use of UHPLC-DAD in this work has allowed to complete the analysis of each run in 14 min, considerably improving the length of time require for this analysis, and consequently decreasing the limit of detection. This is the first time that the use of UHPLC is being reported for the evaluation of coffee leaves.

All the main compounds under investigation, trigonelline, theobromine, caffeine, 3-CQA, 5-CQA, 4-CQA, 3,4-DiCQA, 3,5-DiCQA, 4,5-DiCQA, mangiferin, and isomangiferin, were successfully separated and identified by UHPLC-DAD and their structure confirmed via MS/MS analyses of specific transitions (Table S2). The quantification of alkaloids, CGAs and xanthones in the dry coffee leaf samples was done by UV-Vis spectroscopy, using the DAD detector, by monitoring the absorbance at the maximum wavelength for each compounds, i.e., 254 nm for trigonelline, 258 nm for mangiferin and isomangiferin, 273 nm for theobromine and caffeine and 324 nm for the CGA. Calibration curves were prepared in the range of concentration comprised between 4 and 200 µg/mL and the data were fitted using least-square linear regression. Good linearity (*r*^2^ > 0.9987), expressed as the determination coefficient, was obtained for all compounds, as shown in Table 1.

The limit of detection (LOD) and the limit of quantification (LOQ), the minimum amount of an analyte in a sample that can be detected and quantified are important parameters for the comparison of different sensitivities of analytical methods. However, literature data on the quantification of main secondary metabolites present in coffee leaves do not often provide this information. To the best of our knowledge only one study has reported values of LOD and LOQ when quantifying mangiferin in coffee leaves [12]. 

The quantification method used in this work provided values of LODs for all analytes, and these were found to be ranging from 0.003 to 0.110 µg/mL, which are considerably lower than the concentrations reported until now in literature, for coffee leaf samples [10,11,12]. Comparison of the LOD data can only be done with metabolites isolated from different matrices, other than coffee leaves. Trigonelline showed comparable LOD (0.11 mg/L) to a HPLC-MS method used to analyze roasted and green coffee beans [29]. For caffeine and theobromine, the LODs were found to be the lowest, in the range of 0.003–0.023 µg/mL, which are consistent with the one reported for caffeine in coffee beans determined by HPLC-MS [29]. The LODs for CGAs were in the range of 0.044–0.108 µg/mL, similar to the values obtained when brewed coffee was analyzed using a HPLC-DAD method [30]. The LODs of mangiferin and isomangiferin (0.025 mg/L) were found to be at least 50 times lower than the values reported for HPLC-UV method used to analyse coffee leaves [12], proving the higher sensitivity of UHPLC over HPLC.

The intra-assay (intra-day) and inter-assay (inter-day) variability of the method are parameters that evaluate the accuracy and precision of an analytical method, which is characterized by the closeness between the measured values obtained by replicate measurements. In this study, the intra- and inter-assay variability were assessed by analyzing the aqueous standards of the analytes of interest. The precision was evaluated through intra-day and inter-day relative standard deviation percentage (RSD) and percent bias values for three different concentration levels, as shown in Table S3. The results showed an RSD lower than 2.98% for the intra- and inter-assay for all the analytes, consistent with literature data [12,29,30]. The percent bias value lower than 15% (absolute value) was verified for all the analytes, with the exception of 4,5-DiCQA at the lowest concentration (−19.89% and −21.39%, intra and inter-assay respectively). These data confirm the precision and accuracy of the developed analytical method, which follows the required acceptance criteria of RSD < 15%, and the percent bias value within ±15%.

### 3.2. Quantification of Alkaloids, Chlorogenic acids, Mangiferin, and Isomangiferin in Coffee Leaves

**Figure 1:**
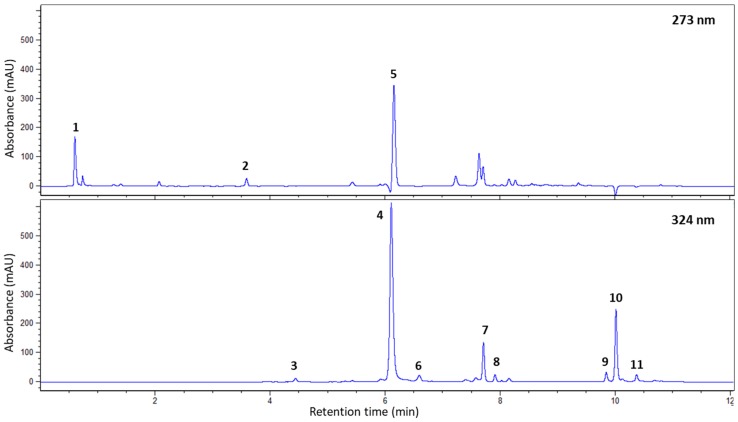
This UHPLC profile of *a C. arabica* young leaf extract. Absorption profile obtained at 273 and 324 nm using the technical conditions for quantitative analysis. Peak 1 = trigonelline; Peak 2 = theobromine; Peak 3 = 3-caffeoylquinic acid (3-CQA); Peak 4 = 5-caffeoylquinic acid (5-CQA); Peak 5 = caffeine; Peak 6 = 4-caffeoylquinic acid (4-CQA); Peak 7 = mangiferin; Peak 8 = isomangiferin; Peak 9 = 3,4-dicaffeoylquinic acid (3,4 diCQA); Peak 10 = 3,5-dicaffeoylquinic acid (3,5-diCQA); Peak 11 = 4,5-dicaffeoylquinic acid (4,5-diCQA).

The UHPLC-DAD quantitative method described here allowed the separation of chlorogenic acids (CQAs, diCQAs, *p*-CoQAs, FQAs, and the correspondent *cis* isomers of CQAs, *p*-CoQAs, and diCQAs), alkaloids (caffeine, theobromine, and trigonelline), mangiferin and isomangiferin (Figure 1). Trigonelline, theobromine, caffeine, mono- and di-chlorogenic acids, mangiferin, and isomangiferin were quantified using the calibration curves previously described (Table 1). FQAs, *p*-CoQAs and *cis* isomers were identified by UHPLC-MS/MS analysis. Analysis by ESI-MS/MS was performed only for qualitative purposes, due to lack of available commercial or synthesized standards of certified purity (Table S2). The MRM transitions used to identify the different minor CGAs have been previously reported [31].

#### 3.2.1. Alkaloids

Trigonelline, a pyridine alkaloid, was found in all the coffee leaf species analysed (Table 2). Its content in *C. arabica* young leaves (11.718 mg/g of dry matter) is in accordance with previous reported data (10.990 mg/g of dry matter) [32]. Young leaves of both genotypes of *C. racemosa* and *C. eugenioides* showed also a trigonelline content (11.188 and 11.090 mg/g and, 14.596 and 9.932 mg/g of dry matter, respectively) similar to that of *C. arabica* young leaves, while *C. canephora* young leaves showed a lower content in trigonelline (7.879 and 4.817 mg/g of dry matter). The mature leaves showed a decrease of trigonelline content in comparison to young leaves, ranging from 12.1% to 24.1%, in *C. canephora*, *C. eugenioides*, and *C. racemosa*, which is in agreement with 6-month-old *C. arabica* seedlings previously reported (up to 23.6%) [32]. 

The content of trigonelline in young leaves, ranging from 0.48% to 1.46% on dry matter basis (dmb) is similar to the content found in the green coffee beans of the species studied [33]. This evidence suggests that a coffee leaf tea would contain at least double the amount of trigonelline, considered a novel phytoestrogen in coffee beans [22], compared to the average quantity present in a coffee beverage; this can be easily justified as up to 70% of its content is lost during the roasting process to form compounds that are responsible for both flavour and bioactivity of coffee [34].

Caffeine and theobromine were the only purine alkaloids only found in three species of coffee leaves, namely *C. arabica, C. canephora*, and *C. racemosa.* The absence of the theophylline in coffee leaves is in accordance with previous studies [10,35], as the catabolism of caffeine is very low, leading to loss of caffeine in coffee leaves. Caffeine was only detected in the leaves of *C. arabica* and *C. canephora*, with concentrations of 14.936 mg/g and 10.043–13.186 mg/g (young leaves) and 5.993 mg/g and 2.805–7.527 mg/g (mature leaves) of dry matter respectively (Table 2). The concentrations of caffeine in young leaves are consistent with previous findings [36] and comparable to the amount present in *C. arabica* green coffee beans [33]. Although the similar concentration of caffeine found in both *C. arabica* and *C. canephora* leaves, these are known to differ considerably between green/roasted *C. arabica* and *C. canephora* beans, a higher variability in the caffeine content of *C. canephora* than in *C. arabica* has been often reported as a result of different genotypes and/or climatic conditions [33,37]. When mature leaves were analyzed, a decrease in caffeine was observed, ranging between 43% and 72%, which was proportional to the decrease in caffeine content of *C. arabica* leaves observed by Ashihara et al. [10]. Although more recently a slow rate of caffeine catabolism was observed, the fact that caffeine biosynthesis occurs mainly in young leaves may explain the variation observed in mature leaves [38]. The presence of very small amounts of caffeine in our samples of *C. eugenioides* and *C. racemose* leaves (inferior to the LOD, determined for the UHPLC-DAD method) could be the consequence of a rapid catabolism of caffeine accompanied by a slow rate of caffeine biosynthesis in these species. 

Theobromine, was also found only in *C. arabica* and *C. canephora* and in one genotype of *C. racemosa* (Table 2). Young leaves showed a theobromine content of 0.811 mg/g of dry matter in *C. arabica*, half of the content reported in a previous study in fresh matter [10]. In *C. canephora* young leaves, a high variability found on the theobromine content (0.169–4.142 mg/g of dry matter) between the two genotypes studied has been also reported [35]. In mature leaves, a decrease of 94% of the theobromine content was verified for *C. arabica*, which has been previously observed by Ashihara et al. [10], and a decrease ranging from 73% to 81% of the theobromine in *C. canephora* which is comparable to the decrease verified in *C. arabica* leaves. In the low caffeine content species, *C. racemosa* and *C. eugenioides*, theobromine was surprisingly found in moderate amounts (1.53 mg/g of dry matter) in the leaves of one genotype of *C. racemosa*, once has been reported the absence of this metabolite in *C. racemosa* leaves [39]. A decrease of theobromine content with leaf aging was also observed in *C. racemose* leaves.

As a result of this thorough analysis, it can be concluded that teas produced from the coffee leaves of *C. eugenioides* and *C. racemosa* can be considered naturally decaffeinated products. The low content of caffeine detected (<0.1% on a dry basis), in accordance with the current accepted coffee regulations [40], allows such labelling without the need for a decaffeination process. This is significant, as it would impact directly the economics of coffee production, reducing considerably the price of this type of product for people who are sensitive to caffeine or wish to limit its intake.

#### 3.2.2. Chlorogenic Acids

As shown in Table 3, the total CGAs content in young leaves, calculated as the sum of mono- and di-caffeoylquinic acids, varied from 35.732 to 80.836 mg/g of dry matter; *C. arabica* and *C. eugenioides* showed the highest content, while *C. canephora* and *C. racemosa* showed the lowest content in CGAs, consistent with the trend recently reported by Campa et al. [11]. The overall high content of CGAs in young coffee leaves is in agreement with previous studies on different coffee leaf species [11,13,16,17]. Specifically, a high content of 5-CQA followed by 3,5-DiCQA (2 to 8 times lower than 5-CQA) was also found, the sum of which accounts for 82% of the total amount of CGAs present in coffee leaves; this trend had already been reported [13,16,17]. However, in young *C. racemosa* leaves a lower content 3,5-DiCQA was observed, while 3-CQA and 4-CQA contents were considerably higher than the values obtained for the young leaves of the other coffee species analysed in this study.

The mature leaves showed a decrease in concentration of CGAs that ranged from 40.8% to 51.7% less than the concentration in their respective young leaves, with the exception of *C. racemosa* CIFC 1693/76 which showed a lower decrease in the concentration of CGAs of (27.4%) and *C. arabica* Bourbon which showed a higher decrease in the concentration of CGAs (78%) with aging (Table 3). More specifically, the decrease in 5-CQA content observed ranged from 26.3% to 48.5%, with the exception for *C. arabica* leaves (74.2%), while the decrease in 3,5-DiCQA content observed was almost the double (46.3%–97.2%). The decrease in content of the other CGAs was also observed but to lesser extent, 12.5% to 31.9% less than in young leaves. These data show that 3,5-DiCQA is the compound whose concentration is most susceptible to aging, followed by 5-CQA, which was also previously verified [16,17], and the other chlorogenic acids present in a less extent, suggesting that CQAs and DiCQAs might undergo different metabolic processes of degradation during leaf aging. The same pattern in change of the concentration with of the different CGAs was also verified in the whole fruit and pericarp of *C. arabica* and *C. canephora* [41]. Interestingly, in *C. arabica* var. Bourbon, the content of 3-CQA and 4-CQA increased with aging, 62% and 17% respectively, as recently reported by Campa et al. [17].

The quantitative comparison of the content of CGAs of coffee leaf samples from different studies is often challenging, as the biosynthesis of these secondary metabolites is not only dependent on the age of the plant from which the leaves were collected but also on the environmental/greenhouse conditions. A recent study demonstrated a significant variation of the concentration of the different CGAs in *C. arabica* leaves originating from different regions in Brazil [13]. This highlights the importance of citing the environmental context in which samples are grown and their development stage when reporting quantitative analysis of coffee leaves. Nevertheless, a general trend can be observed within literature data, in which 5-CQA results as the most abundant chlorogenic acid, followed by 3,5-DiCQA and the rest of CGAs, namely 3-CQA, 4-CQA, 3,4-DiCQA, and 4,5-DiCQA [13,16].

A number of other chlorogenic acids, present in very low concentration, were also identified by ESI-MS/MS (Table S2) due to the absence of HPLC standards; these were 3-*p*-coumaroylquinic acid (3-*p*CoQA), 4-*p*-coumaroylquinic acid (4-*p*CoQA), 5-*p*-coumaroylquinic acid (5-*p*CoQA), 4-feruoylquinic acid (4-FQA), 5-feruoylquinic acid (5-FQA), *cis* 3-caffeoylquinic acid (*cis* 3-CQA), *cis* 3-*p*-coumaroylquinic acid (*cis* 3-pCoQA), *cis* 4-caffeoylquinic acid (*cis* 4-CQA), *cis* 4-*p*-coumaroylquinic acid (*cis* 4-pCoQA), *cis* 5-caffeoylquinic acid (*cis* 5-CQA), *cis* 5-*p*-coumaroylquinic acid (*cis* 5-pCoQA), and one of the *cis* isomers of 3,5-DiCQA. The presence of these minor CGAs and the absence of 3-feruoylquinic acid (3-FQA) has been previously observed [11]. Particularly, the presence of *cis* the isomers of CGAs, which are present in very low concentrations in coffee beans, is expected as coffee leaves are naturally more exposed to ultraviolet (UV) light compared to coffee beans. However, the concentration of *cis* isomers of CGA present in all the analysed samples was too low to allow their quantification, possibly due to the limited exposure of coffee leaves to UV light, as a result of being grown inside greenhouses [11].

The total amount of CGAs accumulated in the young leaves (dry matter) was found to be 7.4% in *C. arabica*, 3.6% and 4.7% in *C. canephora*, 4.4% and 6.2% in *C. eugenioides*, and 4.1% and 8.1% in *C. racemosa*. It is noteworthy that *C. canephora, C. eugenioides*, and *C. racemosa*, showed quantities of CGAs accumulated in their young leaves similar to the amounts present in green coffee beans with the exception of *C. canephora* young leaves, which showed half of the quantity of CGAs present in its coffee beans, as previously observed for caffeine [42].

The results suggest that coffee leaf tea could have health benefits comparable to green coffee beans due to the similar concentrations of CGAs, which are twofold higher than in roasted coffee. During roasting a significant portion of these compounds are transformed into derivatives that are responsible for coffee flavour [34]. *C. eugenioides* or *C. racemosa* have very low bean yield and therefore are less economically relevant species in coffee production; for this reason, they could be used instead for coffee leaf tea preparation, without compromising the coffee bean process of more economically relevant species, such as *C. arabica* and *C. canephora*.

In traditional Chinese medicine the flower and buds of *Lonicera japonica* (Japanese honeysuckle) and, the leaves of *Eucommia ulmoides* are widely used for their beneficial biological properties, due to a high concentration of CGAs present in these plant materials [43]. In fact, chlorogenic acid has been used in Chinese Pharmacopeia as a biomarker to characterise the quality of *Lonicera* flower in terms of biological activities, making it the main active compound responsible for the beneficial biological properties [44]. The concentration of CGAs found in *L. japonica* ranges from 20 to 60 mg/g of dry matter depending on the habitat [43], which is comparable to the concentration of CGAs found in all the coffee leaf species analysed here. Therefore, from an application point of view, coffee leaf tea should provide similar health benefits.

#### 3.2.3. Xanthones

Both mangiferin and isomangiferin were only found in *C. eugenioides* and *C. arabica* leaves. Young leaves showed a mangiferin content of 14.714 mg/g of dry matter in *C. arabica* leaves and, 53.875 and 76.686 mg/g of dry matter in *C. eugenioides* (Table 4), which are comparable to the levels of mangiferin detected in the leaves of mango trees [20], one of the main sources of mangiferin. As in the case of CGAs content, also here a quantitative comparison of the content of xanthones among different studies is challenging, as a high variability has been observed among different samples of *C. arabica* leaves from different regions in Brazil and Costa Rica, suggesting that biosynthesis of these secondary metabolites is largely dependent on environmental conditions [12,13]. For instance, the mangiferin content presently reported for young *C. arabica* leaves was found to be at least two times higher than the mangiferin content reported by Campa et al. [11] and de Almeida et al. [13], and from two times higher to three times lower than the mangiferin content reported by Trevisan et al. [12]. However, the concentration of mangiferin found on *C. eugenioides* leaves by Campa et al. [11] is comparable to the concentration of mangiferin found in this study.

The identification of isomangiferin in the ethanol extracts, which display the typical UV spectrum of mangiferin, was determined from the mass spectrum which showed the same fragmentation pattern of its corresponding isomer, mangiferin. The quantification of isomangiferin was also performed using the calibration curve of mangiferin from the UHPLC-UV chromatograms at 258 nm. Young leaves showed an isomangiferin content of 1.076 mg/g of dry matter in *C. arabica* leaves, 20% more than the content verified by Trevisan et al. [12], and 9.64 mg/g and 12.96 mg/g of dry matter in *C. eugenioides* (Table 4). Additionally, the concentration of isomangiferin was six times lower than mangiferin, which was also verified by Trevisan et al. [12].

The content of mangiferin and isomangiferin was also analysed during leaf aging, which so far has been only studied in *C. arabica* leaves by Trevisan et al. [12]. The aging process led to a decrease in the mangiferin and isomangiferin content of about 85% and 90%, respectively, in *C. arabica* leaves, which was higher than the decrease previously reported [12]. The *C. eugenioides* mature leaves showed a decrease in the concentration for both xanthones of 44% in CIFC 1634/11 variety, and 58% for CIFC 241/43 variety, in comparison to their concentration in young leaves (Table 5). Interestingly, the decrease of both xanthones showed to be comparable to catabolism rates of 5-CQA and 3,5-DiCQA in both coffee species. This observation suggests that most probably, due to the presence of the catechol moiety, mangiferin is catabolized by the same enzymes responsible for the degradation of CGAs in coffee.

These xanthones, which possess high potential as cancer chemopreventive, antioxidant and anti-inflammatory agents [45], are found in high concentrations in *M. indica* (mango) leaves and *Cyclopia genistoides* (honeybush) shoots, the latter being widely used in South Africa to prepare infusions and with a growing worldwide market [19]. Honeybush tea is known for its beneficial biological properties demonstrated both in vivo and in vitro models, as a result of the presence of high concentrations of mangiferin [19]. The concentration of mangiferin found in *C. genistoides* shoots, up to 72.1 mg/g of dry matter, was found to be comparable to the young leaves of *C. arabica* and *C. eugenioides* [19], suggesting that a coffee leaf tea would contain an amount of mangiferin comparable to the one present in honeybush tea, in addition to the high concentration of CGAs in coffee leaves.

The data confirm how an infusion made from young coffee leaves would provide a beverage rich in polyphenols, with potential benefits to human health. Recently, the effect of different tea processing methods on young and mature coffee leaves has been studied and their bioactivities evaluated [25,26]. On one hand, the high content in polyphenols (mainly 5-CQA, 3,5-DiCQA and mangiferin), present in young leaves (*C. arabica*), is responsible for its high antioxidant and anti-inflammatory activities. On the other hand, the mature leaves (*C. arabica*) do not shown such a high antioxidant and anti-inflammatory activities, due to the lack of polyphenols, but can potentially diminish high blood pressure and protect against microbial invasion [25,26]. 

## 4. Conclusions

The present study reports the content of a number of bioactive compounds (trigonelline, xanthines, CGAs, and xanthones) abundant in four different coffee leaf species at different development stages: *C. arabica, C. canephora, C. eugenioides*, and *C. racemosa*. Trigonelline, recently considered a novel phytoestrogen in coffee beans, was found in the leaves of all four different coffee species analyzed. In particular, young leaves contain a concentration of trigonelline comparable to the one found in green coffee beans, which means that a coffee leaf tea would contain at least double the amount of trigonelline present in coffee, due to its partial degradation during roasting. Caffeine was found in the young leaves of *C. arabica* and *C. canephora* in similar concentrations to the ones found in the green coffee beans of *C. arabica*, while the quantification in *C. eugenioides* and *C. racemosa* proved to be very challenging. A coffee leaf tea produced from *C. eugenioides* and *C. racemosa* provides a naturally decaffeinated product potentially useful for consumers’ sensitive to caffeine. The last alkaloid found in abundance in coffee leaves was theobromine, which was present in *C. arabica* and *C. canephora* with a high variability (0.17–4.14 mg/g of dry matter). Surprisingly, theobromine was found in a moderate amount in the leaves of one of the two *C. racemosa* genotypes (CIFC 1693/76).

All the young leaves of coffee species analyzed showed the presence of CGAs in similar quantities to those that are found in the green coffee beans of their respective species, with the exception of *C. canephora* that showed half of the CGAs present in green coffee beans. The most abundant CGAs in the majority of the coffee leaf species were 5-CQA and 3,5-DiCQA, which were also the most affected during leaf aging through possibly different metabolic processes of degradation. From an application point of view, a coffee leaf tea shares the same beneficial biological properties present in green coffee beans, due to the high content of CGAs, higher than in coffee beverages. Xanthones (mangiferin and isomangiferin), which haven’t been found in other coffee parts, were found only in *C. arabica* and *C. eugenioides* and their young leaves showed high and comparable amounts to those found in the leaves of mango tree and honeybush shoots. In both species, the xanthones content was highly affected during leaf aging, as observed for 5-CQA and 3,5-DiCQA.

Recently, it was proved that tea made from the coffee leaves of *C. arabica* have strong antioxidant and anti-inflammatory properties, mainly due to the presence of phenolic compounds, in particular 5-CQA and mangiferin, which are the two most abundant [25,26]. The present study suggests that the leaves of *C. eugenioides* are more promising than *C. arabica* leaves to produce coffee tea leaf, mainly due to their low economic relevance (low beans yield) and higher content in polyphenols, more specifically in mangiferin. Additionally, *C. eugenioides* leaves are able to produce a beverage which is naturally decaffeinated, reducing considerably the price for people who are sensitive to caffeine or wish to limit its intake. Therefore, further research on the potential benefits of a coffee leaf tea made from *C. eugenioides* leaves would be desirable.

The data overall confirm that indeed coffee leaf tea has the phytochemical profile to be used in the food industry as an alternative to tea, and that the choice of botanical species and their degree of maturity needs to be carefully evaluated, given their impact on the concentration of bioactive compounds.

## Figures and Tables

**Table 1:** Ultra-high-performance liquid chromatography (UHPLC)-UV method optimization results.

Analyte	λ_max_ (nm)	Retention Time (min)	Calibration Curve			LOD ^1^ (µg/mL)	LOQ ^2^ (µg/mL)
			Intercept	Slope	*r* ^2^		
Trigonelline	254	0.60	9.025	5.375	0.9996	0.110	0.365
Mangiferin Isomangiferin	258	7.71 7.85	4.986	16.409	0.9987	0.025	0.083
Theobromine	273	3.65	1.482	17.420	0.9999	0.023	0.075
Caffeine		6.24	32.541	14.371	0.9999	0.003	0.012
3-CQA	324	4.49	8.217	8.924	0.9999	0.066	0.220
5-CQA		6.18	23.912	8.487	0.9998	0.089	0.297
4-CQA		6.66	0.710	9.330	0.9999	0.044	0.145
3,4-DiCQA		9.88	1.354	8.913	0.9995	0.016	0.054
3,5-DiCQA		10.05	0.771	11.102	0.9999	0.108	0.359
4,5-DiCQA		10.41	−1.908	11.486	0.9993	0.057	0.190

^1^ LOD = Limit of detection, ^2^ LOQ = Limit of quantification.

**Table 2:** Alkaloids composition of coffee leaves (mg/g of dry matter ± SD).

Species	Coffee Genotype	Trigonelline		Theobromine		Caffeine *		Total Alkaloids	
		Young	Mature	Young	Mature	Young	Mature	Young	Mature
***C. arabica***	var. Bourbon	11.718 ± 0.544	2.955 ± 0.207	0.811 ± 0.029	0.045 ± 0.005	14.936 ± 0.019 **a**	5.993 ± 0.508 **e**	27.465 ± 0.592	8.993 ± 0.720
***C. canephora***	CIFC 2975	7.879 ± 0.209	5.979 ± 0.017	0.169 ± 0.001	0.046 ± 0.000	13.186 ± 0.423 **b**	7.527 ± 0.032 **d**	21.234 ± 0.633	13.552 ± 0.049
	CIFC 829/1	4.817 ± 0.019	4.157 ± 0.300	4.142 ± 0.040	0.806 ± 0.076	10.043 ± 0.043 **c**	2.805 ± 0.077 **f**	19.002 ± 0.102	7.769 ± 0.454
***C. eugenioides***	CIFC 1634/11	14.596 ± 0.143	11.232 ± 0.001	-	-	-	-	14.596 ± 0.143	11.232 ± 0.001
	CIFC 241/43	9.932 ± 0.021	7.781 ± 0.002	-	-	-	-	9.932 ± 0.021	7.781 ± 0.002
***C. racemosa***	CIFC 1693/76	11.188 ± 0.222	9.836 ± 0.177	1.532 ± 0.026	0.549 ± 0.003	-	-	12.721 ± 0.245	10.385 ± 0.180
	CIFC 13969	11.090 ± 0.245	9.736 ± 0.092	-	-	-	-	11.090 ± 0.245	9.736 ± 0.092

Data are shown as means ± SD. Total alkaloids are the sum of trigonelline, theobromine and caffeine. * Total caffeine concentrations are labelled with different letters (a–f) to highlight values that are considered statistically different (*p* < 0.5).

**Table 3:** Chlorogenic acids (CGAs) composition of coffee leaves (mg/g of dry matter ± SD).

Species	Coffee Genotypes	3-CQA		5-CQA		4-CQA		3,4-DiCQA	
		Young	Mature	Young	Mature	Young	Mature	Young	Mature
***C. arabica***	var. Bourbon	0.881 ± 0.070	1.429 ± 0.103	56.790 ± 3.640	14.678 ± 1.617	1.586 ± 0.110	1.853 ± 0.155	1.798 ± 0.133	0.431 ± 0.040
***C. canephora***	CIFC 2975	0.906 ± 0.031	0.873 ± 0.002	22.880 ± 0.796	13.874 ± 0.033	0.906 ± 0.006	1.035 ± 0.008	1.003 ± 0.007	0.441 ± 0.000
	CIFC 829/1	1.395 ± 0.002	1.217 ± 0.031	29.458 ± 0.155	16.346 ± 0.798	3.850 ± 0.003	2.602 ± 0.175	1.001 ±0.007	0.498 ± 0.018
***C. eugenioides***	CIFC 1634/11	0.521 ± 0.003	0.402 ± 0.004	49.581 ± 0.299	31.451 ± 0.538	3.148 ± 0.000	2.872 ± 0.040	0.549 ± 0.001	0.257 ± 0.005
	CIFC 241/43	0.716 ± 0.006	0.450 ± 0.005	34.283 ± 0.657	17.667 ± 0.200	5.096 ± 0.039	3.388 ± 0.040	0.421 ± 0.012	0.044 ± 0.000
***C. racemosa***	CIFC 1693/76	3.007 ± 0.030	2.816 ± 0.018	21.123 ± 0.129	15.574 ± 0.002	8.359 ± 0.112	6.546 ± 0.099	0.797 ± 0.009	0.628 ± 0.032
	CIFC 13969	1.618 ± 0.016	2.234 ± 0.006	46.145 ± 0.752	24.781 ± 0.129	8.232 ± 0.050	6.282 ± 0.032	2.044 ± 0.101	1.768 ± 0.043
**Species**	**Coffee Genotypes**	**3,5-DiCQA**		**4,5-DiCQA**		Total CGAs *	
		**Young**	**Mature**	**Young**	**Mature**	**Young**	**Mature**
***C. arabica***	var. Bourbon	11.266 ± 0.591	0.318 ± 0.030	1.217 ± 0.045	0.499 ± 0.067	73.539 ± 4.590 **b**	19.208 ± 2.012 **i**
***C. canephora***	CIFC 1693/76	9.952 ± 0.184	2.994 ± 0.034	0.085 ± 0.001	0.095 ± 0.003	35.732 ± 1.027 **g**	19.312 ± 0.080 **i**
	CIFC 13969	11.035 ± 0.070	1.934 ± 0.019	0.185 ± 0.003	0.066 ± 0.013	46.924 ± 0.239 **d**	22.663 ± 1.042 **i**
***C. eugenioides***	CIFC 2975	6.513 ± 0.136	3.495 ± 0.302	0.704 ± 0.118	0.344 ± 0.019	40.503 ± 0.533 **ef**	29.402 ± 0.473 **h**
	CIFC 829/1	20.309 ± 0.423	6.739 ± 0.001	2.488 ± 0.080	2.308 ± 0.105	80.836 ± 1.422 **a**	44.112 ± 0.180 **de**
***C. racemosa***	CIFC 1634/11	6.837 ± 0.065	1.348 ± 0.011	1.150 ± 0.013	0.272 ± 0.003	61.782 ± 0.382 **c**	36.603 ± 0.600 **fg**
	CIFC 241/43	2.388 ± 0.068	0.084 ± 0.000	0.914 ± 0.033	0.091 ± 0.003	43.818 ± 0.814 **de**	21.724 ± 0.248 **i**

Data are shown as means ± SD. Total CGAs are the sum of 3-, 4-, 5-CQA and 3,4-; 3,5-; 4,5-diCQA. * Total CGAs concentrations are labelled with letters (a–i) that group values that are considered not statistically different (*p* > 0.5).

**Table 4:** Xanthones composition of coffee leaves (mg/g of dry matter ± SD).

Species	Coffee Genotypes	Mangiferin *		Isomangiferin		Total Xanthones	
		Young	Mature	Young	Mature	Young	Mature
***C. arabica***	var. Bourbon	14.714 ± 0.838 **e**	2.222 ± 0.326 **f**	1.076 ± 0.038	0.111 ± 0.002	15.789 ± 0.876	2.333 ± 0.328
***C. canephora***	CIFC 2975	-	-	-	-	-	-
	CIFC 829/1	-	-	-	-	-	-
***C. eugenioides***	CIFC 1634/11	53.875 ± 0.168 **b**	30.195 ± 0.714 **d**	9.644 ± 0.026	5.392 ± 0.128	63.519 ± 0.194	35.587 ± 0.842
	CIFC 241/43	76.686 ± 1.176 **a**	32.605 ± 0.081 **c**	12.959 ± 0.335	5.411 ± 0.147	89.645 ± 1.511	38.016 ± 0.228
***C. racemosa***	CIFC 1693/76	-	-	-	-	-	-
	CIFC 13969	-	-	-	-	-	-

Data are shown as means ± SD. Total xanthones are the sum of mangiferin and isomangiferin. * Total mangiferin concentrations are labelled with different letters (a–f) to highlight values that are considered statistically different (*p* < 0.5).

**Table 5:** Decrease of the concentration of 5-CQA, 3,5-DiCQA, and xanthones during leaf aging.

Species	Coffee Genotypes	Decrease during Leaf Aging (%)		
		5-CQA	3,5-DiCQA	Xanthones
***C. arabica***	var. Bourbon	74	97	85
***C. eugenioides***	CIFC 241/43	37	46	44
	CIFC 1634/11	48	67	58

## Supplementary Materials

The following are available online at https://www.mdpi.com/2076-3921/9/1/6/s1, Table S1: Dimension of fresh leaves (cm) of the different *Coffea* species at different development stages, Table S2: Electrospray ionization mass–spectrometry characterization of isomers, Table S3: Intra- and interday precision and accuracy of the HPLC standards of chlorogenic acids (CGAs), alkaloids and mangiferin.
