# Supplementary material for: Dietary Antioxidants in Coffee Leaves: Impact of Botanical Origin and Maturity on Chlorogenic Acids and Xanthones
Source: Antioxidants (Basel). 2019 Dec 20;9(1):6. doi: 10.3390/antiox9010006 (PMC7023256; doi:10.3390/antiox9010006)
Supplement: Supplementary file 1 [file antioxidants-09-00006-s001.pdf]

antioxidants

Supplementary data

Dietary antioxidants in coffee leaves: impact of botanical origin and maturity on chlorogenic acids and xanthones

Ângelo Monteiro [1,*], Silvia Colomban [2], Helena Azinheira [3,4], Leonor Guerra-Guimarães [3,4], Maria Do Céu Silva [3,4], Luciano Navarini [2] and Marina Resmini [1,*]

1 Department of Chemistry, Queen Mary University of London, Mile End Road, London E1 4NS, United Kingdom; a.f.monteiro@qmul.ac.uk (A.M.); m.resmini@qmul.ac.uk (M.R.)
2 illycaffè S.p.A., via Flavia 143, 34100 Trieste, Italy; silvia.colomban@qmul.ac.uk (S.C.); luciano.navarini@illy.com (L.N.)
3 Centro de Investigação das Ferrugens do Cafeeiro, Instituto Superior de Agronomia, Universidade de Lisboa, Oeiras, Portugal; hgazinheira@isa.ulisboa.pt (H.A.); leonorguimaraes@edu.ulisboa.pt (LGG); mariaceusilva@isa.ulisboa.pt (M.C.S.)
4 Linking Landscape, Environment, Agricultural and Food, Instituto Superior de Agronomia, Universidade de Lisboa, Lisboa, Portugal; hgazinheira@isa.ulisboa.pt (H.A.); leonorguimaraes@edu.ulisboa.pt (LGG); mariaceusilva@isa.ulisboa.pt (M.C.S.)
* Correspondence: a.f.monteiro@qmul.ac.uk (A.M.); m.resmini@qmul.ac.uk (M.R.)

Table S1. Dimension of fresh leaves (cm) of the different *Coffea* species at different development stages.

Maturation	Species	Genotypes	Length (cm)	Width (cm)
**Young**	*C. arabica*	var. Bourbon	7.6 ± 0.9	3.3 ± 0.4
	C. canephora	CIFC 829/1	12.6 ± 1.2	6.2 ± 0.8
		CIFC 2975	13.6± 0.8	7.2 ± 0.7
	C. eugenioides	CIFC 241/43	6.4 ± 1.0	2.7 ± 0.3
		CIFC 1634/11	7.1 ± 1.1	2.8 ± 0.6
	C. racemosa	CIFC 1693/76	10.3 ± 1.0	5.3 ± 0.7
		CIFC 13969	11.1± 0.8	6.1 ± 0.6
**Mature**	*C. arabica*	Bourbon	16.6 ± 1.9	7.5 ± 0.8
	C. canephora	CIFC 829/1	19.3 ± 2.5	9.9 ± 1.1
		CIFC 2975	18.7 ± 0.7	9.3 ± 0.6
	C. eugenioides	CIFC 241/43	9.8 ± 0.7	4 ± 0.2
		CIFC 1634/11	10.0 ± 0.6	4.1 ± 0.3
	C. racemosa	CIFC 1693/76	17.1 ± 2.1	9.1 ± 1.0
		CIFC 13969	18.2 ± 2.2	9.9 ± 0.9

Table S2. Electrospray ionization mass–spectrometry characterization of isomers.

Peak	RT (min)	ESI-MS fragments (m/z)	[M-H]- (m/z)	Abbreviation	Identification
1	4.21	353	**191**, 179	cis 3-CQA	*cis* 3-Caffeoylquinic acid
2*	4.57	353	**191**, 179	3-CQA	3-Caffeoylquinic acid
3	5.55	337	**163**	cis 3-pCoQA	*cis* 3-*p*-Coumaroylquinic acid
4	5.71	337	**163**	3-pCoQA	3-*p*-Coumaroylquinic acid
5	5.77	353	**173**, 179	cis 4-CQA	*cis* 4-Caffeoylquinic acid
6*	6.39	353	**191**, 179	5-CQA	5-Caffeoylquinic acid
7*	6.82	353	**173**, 179	4-CQA	4-Caffeoylquinic acid
8	7.31	337	**173**, 163	cis 4-pCoQA	*cis* 4-*p*-Coumaroylquinic acid
9	7.64	353	**191**, 179	cis 5-CQA	*cis* 5-Caffeoylquinic acid
10	7.86	337	**191**, 179	5-pCoQA	5-*p*-Coumaroylquinic acid
11	7.95	337	**173**, 163	4-pCoQA	4-*p*-Coumaroylquinic acid
12*	7.97	421	**301**	Mangif	Mangiferin
13	8.17	421	**301**	Isomangif	Isomangiferin
14	8.45	367	**191**, 179	5-FQA	5-Feruoylquinic acid
15	8.48	367	**173**, 191	4-FQA	4-Feruoylquinic acid
16	8.63	337	**191**, 179	cis 5-pCoQA	*cis* 5-*p*-Coumaroylquinic acid
17*	10.13	515	**353**	3,4-DiCQA	3,4-Dicaffeoylquinic acid
18*	10.32	515	**353**	3,5-DiCQA	3,5-Dicaffeoylquinic acid
19	10.45	515	**353**	cis 3,5-DiCQA	cis 3,5-Dicaffeoylquinic acid
20*	10.68	515	**353**	4,5-DiCQA	4,5-Dicaffeoylquinic acid

Characterization of chlorogenic acids and xanthonoids (mangiferin and isomangiferin) by UV absorbance spectrum and electrospray ionization-mass spectrometry detection (LC-DAD/ESI-MS). RT, retention time.

* Identified with standard compound.

Table S3. Intra- and interday precision and accuracy polyphenols and alkaloids present in coffee leaves.

Parameters	Value of theoretical concentration (µg / ml) of:																	
	**3-CQA**			**5-CQA**			**4-CQA**			**3,4-DiCQA**			**3,5-DiCQA**			**4,5-DiCQA**		
	**196.7**	**98.7**	**48.5**	**200.7**	**100.7**	**49.5**	**79.7**	**40.0**	**19.7**	**9.6**	**4.8**	**2.4**	**79.3**	**39.8**	**19.6**	**9.6**	**4.8**	**2.4**
**Intraday**																		
**Overall mean (n = 5)**	193.79	98.12	48.00	197.74	100.29	50.14	74.92	38.02	19.17	9.43	4.82	2.25	76.97	39.16	19.01	8.83	4.23	1.90
**SD[1]**	0.15	0.05	0.04	0.10	0.21	0.20	0.05	0.03	0.04	0.02	0.03	0.01	0.11	0.14	0.18	0.05	0.06	0.05
**RSD[2] (%)**	0.08	0.05	0.09	0.05	0.21	0.40	0.07	0.08	0.19	0.22	0.54	0.28	0.14	0.35	0.96	0.61	1.46	2.49
**Bias (%)**	-1.48	-0.58	-1.07	-1.48	-0.41	1.28	-6.02	-4.96	-2.52	-1.87	-0.04	-5.22	-2.96	-1.61	-2.82	-8.19	-12.21	-19.89
**Interday**																		
**Overall mean (n = 15)**	193.72	97.74	47.36	198.42	99.90	49.02	75.09	37.98	19.02	9.47	4.79	2.22	76.67	38.72	18.36	8.89	4.27	1.86
**SD[1]**	0.14	0.33	0.57	0.56	0.28	0.80	0.16	0.03	0.11	0.03	0.03	0.02	0.23	0.34	0.54	0.04	0.03	0.04
**RSD[2] (%)**	0.07	0.34	1.20	0.28	0.28	1.64	0.22	0.08	0.55	0.27	0.64	0.78	0.30	0.88	2.92	0.49	0.76	2.01
**Bias (%)**	-1.51	-0.96	-2.38	-1.14	-0.79	-0.97	-5.81	-5.05	-3.26	-1.52	-0.75	-6.22	-3.34	-2.71	-6.18	-7.55	-11.58	-21.39

	**Trigonelline**			**Theobromine**			**Theophylline**			**Caffeine**			**Mangiferin**		
	191.7	95.7	47.8	16.8	8.4	4.2	22.5	11.2	5.6	198.6	99.2	49.5	17.1	8.4	4.3
**Intraday**															
**Overall mean (*n* = 5)**	190.29	95.11	47.35	16.84	8.46	4.23	22.68	11.21	5.53	197.82	98.22	48.78	17.32	8.45	3.72
**SD[a]**	0.54	0.24	0.06	0.03	0.03	0.01	0.20	0.02	0.01	0.54	0.27	0.09	0.04	0.02	0.01
**RSD[b] (%)**	0.28	0.26	0.13	0.20	0.31	0.17	0.88	0.18	0.21	0.27	0.28	0.19	0.25	0.23	0.19
**Bias (%)**	-0.73	-0.14	-0.89	0.11	0.67	0.94	0.80	0.23	-1.13	-0.39	-0.99	-1.46	1.07	0.58	-14.24
**Interday**															
**Overall mean (*n* = 15)**	191.53	95.54	48.35	16.90	8.49	4.29	22.60	11.20	5.51	198.42	98.53	49.50	17.29	8.41	3.73
**SD[a]**	0.98	0.36	1.36	0.05	0.03	0.09	0.10	0.07	0.02	0.46	0.24	0.93	0.05	0.04	0.08
**RSD[b] (%)**	0.51	0.37	2.81	0.28	0.30	2.05	0.43	0.62	0.34	0.23	0.25	1.88	0.30	0.49	2.21
**Bias (%)**	-0.08	-0.22	1.20	0.51	1.03	2.46	0.43	0.14	-1.48	-0.09	-0.68	-0.01	0.87	0.18	-14.22

[a] SD, standard deviation

[b] RSD, relative standard deviation
